# Preparation of Scalable Silica‐Coated Iron Oxide Nanoparticles for Nanowarming

**DOI:** 10.1002/advs.201901624

**Published:** 2020-01-07

**Authors:** Zhe Gao, Hattie L. Ring, Anirudh Sharma, Baterdene Namsrai, Nam Tran, Erik B. Finger, Michael Garwood, Christy L. Haynes, John C. Bischof

**Affiliations:** ^1^ Department of Mechanical Engineering University of Minnesota 111 Church St. Minneapolis MN 55455 USA; ^2^ Center for Magnetic Resonance Research Department of Radiology University of Minnesota 2021 6th Street S.E. Minneapolis MN 55455 USA; ^3^ Department of Surgery University of Minnesota 420 Delaware Street SE Minneapolis MN 55455 USA; ^4^ Department of Chemistry University of Minnesota 207 Pleasant St SE Minneapolis MN 55455 USA; ^5^ Department of Biomedical Engineering University of Minnesota 111 Church St. Minneapolis MN 55455 USA

**Keywords:** core–shell nanoparticles, cryopreservation, iron oxide nanoparticles, radio frequency warming

## Abstract

Cryopreservation technology allows long‐term banking of biological systems. However, a major challenge to cryopreserving organs remains in the rewarming of large volumes (>3 mL), where mechanical stress and ice formation during convective warming cause severe damage. Nanowarming technology presents a promising solution to rewarm organs rapidly and uniformly via inductive heating of magnetic nanoparticles (IONPs) preloaded by perfusion into the organ vasculature. This use requires the IONPs to be produced at scale, heat quickly, be nontoxic, remain stable in cryoprotective agents (CPAs), and be washed out easily after nanowarming. Nanowarming of cells and blood vessels using a mesoporous silica‐coated iron oxide nanoparticle (msIONP) in VS55, a common CPA, has been previously demonstrated. However, production of msIONPs is a lengthy, multistep process and provides only mg Fe per batch. Here, a new microporous silica‐coated iron oxide nanoparticle (sIONP) that can be produced in as little as 1 d while scaling up to 1.4 g Fe per batch is presented. sIONP high heating, biocompatibility, and stability in VS55 is also verified, and the ability to perfusion load and washout sIONPs from a rat kidney as evidenced by advanced imaging and ICP‐OES is demonstrated.

## Graphical abstract

Nanowarming is a new technology that could solve the technical challenge of volumetric rewarming of cryopreserved biological samples. With the aim of scaling up nanowarming technology from cell and simple tissues to a whole organ, a scalable silica‐coated iron oxide nanoparticle is synthesized and tested for cell nanowarming and organ perfusional loading and removal.

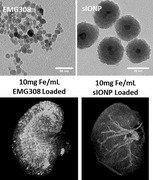

## 1 Introduction

Organ transplantation is often the only curative treatment available for a wide range of end‐stage organ diseases. According to the United Network for Organ Sharing, there are currently over 114 000 people in need of an organ transplant, with somebody new added every 10 min.^1^ Although organ donation has increased in recent years, the number of people on the waiting list continues to grow even faster.^1^, ^2^ The timeframe in which organs can remain viable under static cold storage (the current gold standard preservation method) is very short. Kidneys are viable up to 24–36 h, while hearts and lungs are only viable for 3–6 h.^3^ One‐fifth of donated kidneys and more than 60% of donor hearts and lungs are not used or transplanted in part because their cold ischemic time (i.e., maximum allowable time between recovery from the donor and transplant in the recipient) has been exceeded.^4^ If even half of these discarded organs were available for transplant, wait lists for lungs and heart could be eliminated within 2–3 years.^5^

Cryopreservation, or the preservation of biological samples at cryogenic temperatures (i.e., −150 °C with electronic freezers or −160 °C in nitrogen vapor), is currently the only method that allows for long‐term banking of biological systems. Unfortunately, freezing biological samples with no additional preparation causes cell dehydration and ice formation, resulting in the rupture of cell membranes and vasculature, ultimately resulting in cell and tissue death.^6^ However, with the addition of cryoprotective additives, organs have been vitrified (i.e., chilled to an amorphous phase with no ice crystals) and can theoretically be preserved in this glassy or vitrified state indefinitely.^7^ The availability of such cryogenically banked organs could increase organ transplant utilization, improve short‐ and long‐term graft function, and increase overall patient survival.^4^

Achieving the cryogenic state requires cooling organs fast enough to avoid ice crystal formation. This critical cooling rate (CCR) is a function of the type and amount of cryoprotective agents (CPAs) used. For instance, the CCR for VS55, a commonly used CPA, is −2.5 °C min^−1^, an achievable rate for most systems as shown by Fahy et. al. with rabbit kidneys.^7^, ^8^ However, even if this is achieved, rewarming requires a much faster critical warming rate (CWR), such as 50 °C min^−1^ for VS55[qv: 8c] to avoid devitrification (i.e., ice formation during the rewarming).^9^ Convective heating by immersion in a warm water bath is adequate for samples with volumes below 3 mL and cells in suspension or small tissues in a cryovial or cryobag. However, convective warming is often too slow to achieve needed CWR in larger samples (>3 mL) due to the inability to quickly warm the center of the sample, thereby leading to devitrification. Furthermore, fractures and cracks within the tissue are also caused by thermal stress between the tissue edge and center.^10^

Electromagnetic rewarming of cryopreserved systems at tens to thousands of MHz (including microwave), can be achieved by dielectric heating,^11^ which has been applied with limited success to several systems (Table S1, Supporting Information).^12^ Unfortunately, the strong temperature dependence of the dielectric properties can increase heating in any spot with an elevated temperature (i.e., center, edge, or other) while low thermal conductivity prevents heat spreading thereby leading to “thermal runaway” as a failure mode.[qv: 12d,13] Furthermore, nonuniformity in the field and its absorption (i.e., dissipation, distortion, and shape effects) can be further hindrances to uniform rewarming. For instance, optimal rewarming is predicted for only “small” spheres whose circumference is less than the wavelength of radiation due to the power dissipation and the field distortion.[qv: 13b,14] Moreover, dielectric property variation due to the heterogeneous components in organs^15^ also make it impossible to rewarm vitrified organs solely by electromagnetic heating.[qv: 12b,c] To circumvent these problems, a method that uses heat generation that can be spread sufficiently uniformly in a large system is still needed.

Nanowarming is a new method of volumetric rewarming that theoretically has no size limit and has been demonstrated for volumes as high as 80 mL.^16^ Briefly, nanowarming produces rapid and uniform heating in a sample through inductive heating of iron oxide nanoparticles (IONPs) within a radio‐frequency coil (alternating magnetic field, with frequency of ≈100–400 kHz). With IONPs distributed throughout the sample, rapid and uniform warming occurs, which eliminated cracking and ice formation and results in improved recovery and tissue viability.^16^ We note that hybrid heating methods, such as assisting electromagnetic heating with conduction heating^14^, ^17^ and magnetic nanoparticles^18^ are also being investigated as potential solutions for large‐volume rewarming (Table S1, Supporting Information). However, the success was only limited to large‐volume (20 mL) cells suspensions so far, whereas nanowarming is theoretically fully scalable to L size organ systems.[qv: 16b]

Heat‐producing IONPs are thus an essential component of nanowarming. The heating properties of IONPs have been applied to clinical treatments, such as hyperthermia of glioblastoma and ongoing clinical trials for prostate cancer.^19^ Cancer treatment requires a nanoparticle which is stable in biological fluids (i.e., blood, interstitial, or intracellular), has heating properties tuned to cell destruction, and a low collateral toxicity profile. In some cases, the nanoparticle is even designed for cancer cell uptake.^20^ However, IONPs used for nanowarming need to have different properties than those optimized for cancer treatment. First, they should show minimal cellular association and uptake to allow maximum removal after usage. Second, organ cryopreservation requires the use of CPAs often formulated with organic solvents (i.e., dimethyl sulfoxide, glycols, etc.), sugars, and salts to achieve necessarily low CCRs;[qv: 8b,c] therefore, the IONPs should be colloidally stable in the CPA at high concentration (mg's Fe mL^−1^) such that they can maintain their heating ability and be perfused into the organ and distribute throughout the vasculature as a last step prior to cooling. Once in the vitrified state and sufficiently perfused with IONPs, the organ is theoretically stable for years but can be rewarmed at any point through inductive warming at rates that exceed the CWR of the CPA.^21^

IONPs with varied coatings that have been tested for nanowarming are shown in **Table** **1**.

**Table 1:** IONPs tested for nanowarming

IONP	SAR [W g^−1^ Fe] in H2O^a)^	SAR [W g^−1^ Fe] in VS55^a)^	Stability in VS55	Cytotoxicity in HDF cells	Cellular uptake at 37 °C	Scale‐up to grams
EMG308	409	169	Hours	Starts to show toxicity at 0.5 mg Fe mL^−1^	Yes	Yes
msIONP^22^	309	286	Months	No toxicity shown at 1 mg mL^−1^	No	No
sIONP	392	319	More than 6 months	No toxicity shown at 10 mg Fe mL^−1^	No	Yes

^a)^ SAR was measured at 20 kA m^−1^ and 360 kHz.

EMG308, which demonstrated the first proof of principle of nanowarming in solutions,[qv: 16a] is inexpensive and easily obtained; however, EMG308 is not sufficiently biocompatible, is taken up in cells, and is unstable in CPA. Previously, we coated EMG308 with mesoporous silica (msIONP) to provide stability in CPAs and achieved the first biological demonstration of nanowarming with cells and simple tissues.[qv: 16b] However, msIONP synthesis, aging, deoxygenation, hydrothermal treatment, and purification is laborious and takes several days (Table S2, Supporting Information). Specifically, the elevated reaction temperature and removal of a toxic surfactant, cetyltrimethylammonium bromide (CTAB), from the pores, inhibited scaled‐up production of msIONPs.^22^ One batch of msIONP synthesis could produce 35 mg Fe msIONPs, which is far below the demand in organ nanowarming (Table S3, Supporting Information).

Herein, we report the synthesis and characterization of silica‐coated EMG308 (sIONPs), which eliminated the need for CTAB in synthesis and shortened the overall time frame (Table S2, Supporting Information). Currently, we can produce > 20 g (1.4 g Fe) sIONPs/batch in a 4L reaction vessel, allowing for the scale‐up required for the current rodent organ nanowarming studies (Table S3, Supporting Information). This sIONP production can be further scaled up to larger quantity with a larger reaction vessel.

Our sIONPs were fully characterized using multiple analytical methods. For instance, cores and coating were assessed using transmission electronic microscopy (TEM), dynamic light scattering (DLS), zeta potential, inductively coupled plasma‐optical emission spectroscopy (ICP‐OES), infrared spectroscopy (IR), X‐ray photoelectron spectroscopy (XPS), nitrogen adsorption analysis, and thermal gravity analysis (TGA). Further, the physical properties that are important to nanowarming, especially heating and magnetic properties, were studied as a function of shell thickness and colloidal stability in VS55, a commonly used CPA. Biological assessments included measurement of cytotoxity and cellular uptake in human fibroblasts (HDFs, ATCC) and nanowarming of HDF. Finally, we also present, for the first time, a demonstration that an ex vivo rat kidney can be uniformly loaded and washed out with CPA and sIONPs. sIONP loading and washout were evaluated using microcomputed tomography (µCT) and magnetic resonance imaging (MRI).^23^ In short, we demonstrate that sIONPs are an effective and scalable embodiment of IONPs for nanowarming use in organs.

## 2 Results and Discussion

### 2.1 Silica Coating of EMG308 (sIONP)

EMG308 is a commercially available IONP that heats well in water and is relatively inexpensive.[qv: 16a] However, due to aggregation, the heating ability of EMG308 is significantly lowered in complex media other than water (i.e., saline or protein solutions).^21^ Previous successes in nanowarming of biological samples were performed with msIONPs due to the high stability endowed by their polyethylene glycol (PEG)/trimethyl silane (TMS) coating.[qv: 16b,22] However, the quantity of IONPs required for organ nanowarming is much greater than needed in previous use of nanowarming for arteries or cancer therapeutics (see Table S3, Supporting Information).

#### 2.1.1 sIONP Synthesis and Morphology

The silica shell was coated onto EMG308 using a modified Stöber method.^24^ After the silica shell, the surface was modified with PEG and a small hydrophobic ligand, TMS. PEG is well known as a biocompatible polymer that is antibiofouling and increases nanoparticle circulation time in the body, while the TMS serves as a spacer between the PEG to help fully extend PEG and therefore provide stability in the solutions.^22^, ^25^ Polyvinyl pyrrolidone (PVP) has been demonstrated as a universal surface modifier for coating colloidal particles with silica,^22^, ^26^ so our synthesis used PVP as an intermediate layer for silica coating on EMG308. Although other groups have reported direct silica coating on EMG304,^27^ attempts to produce a silica shell on EMG308 without PVP resulted in free silica and bare EMG308 cores, likely due to the different surfactants on EMG308 versus EMG304 (data not shown).

**Figure 1:**
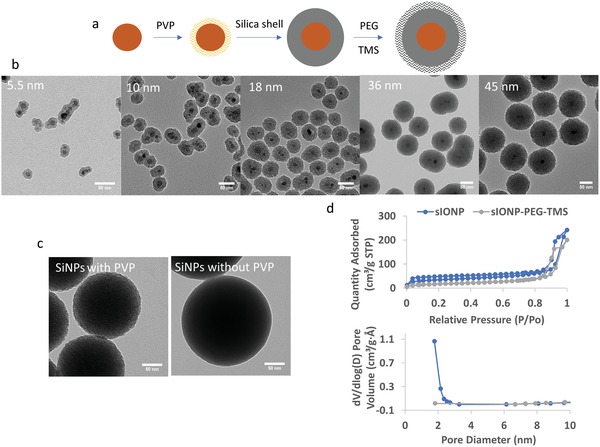
a) Schematic of silica‐coated iron oxide nanoparticle (sIONP) synthesis. b) Representative TEM images of sIONPs with increasing/different silica shell thicknesses. The number on the top left corner indicates the average shell thickness in each image. Scale bar is 50 nm. c) TEM images of silica nanoparticles synthesized with or without PVP. PVP addition causes porosity in silica. Scale bar is 50 nm. d) Nitrogen adsorption analysis data of sIONP with or without surface modification with PEG‐TMS. The isotherm hysteresis loop showed porosity in unmodified sIONPs. The pore size distribution in unmodified sIONP is smaller than 2 nm. The pore of modified sIONP is blocked by PEG.

The scheme for sIONP synthesis is shown in **Figure** **1**a. The silica shell thickness can be easily tuned by varying the amount of silica precursor, tetroethoxysilane (TEOS), added to the reaction. The iron quantification through ICP‐OES shows a linear correlation between the IONP core volume fraction and the iron weight percentage (Figure S1, Supporting Information). The thickest silica shell produced with single‐step TEOS addition was 45 nm (Figure 1b). Excess TEOS addition resulted in free silica in the product (Figure S2a, Supporting Information). Although thicker silica shells could be achieved by multistep addition of TEOS (Figure S2b,c, Supporting Information), the following characterization and application were focused on sIONPs formed with single‐step TEOS addition. EMG308 is naturally polydisperse and contains small IONP agglomerates in solution. When the silica shell was thin, the silica was homogeneously coated on EMG308 cores regardless of their shape and aggregation (Figure 1b), and the resulting sIONPs were relatively polydisperse (polydispersity = 0.170 when shell thickness is 11 nm). When the silica shell was thicker, the core polydispersity was hidden and the resulting sIONPs become more spherical and monodisperse.^27^ However, thicker shells increased the volume occupied per particle and therefore the total Fe that could be suspended in the solution. For instance, sIONPs with 45 nm silica shells could only be concentrated up to 5 mg Fe mL^−1^ in water, but 18 nm silica shells could be concentrated up to 40 mg Fe mL^−1^. Nanowarming applications require high heating rates, so higher Fe concentration solutions are preferred. Thus, we chose sIONPs with 18 nm silica shell thickness which are monodisperse (polydispersity = 0.080), while still allowing high Fe concentration (40 mg Fe mL^−1^) in water. Further surface characterization, scale‐up, and biological experiments were pursued with this 18 nm shell thickness sIONP embodiment.

Similar to the msIONPs, the sIONPs were not all single‐core nanoparticles (as shown in Figure 1b), with around 50% of sIONPs (Figure S3, Supporting Information) multicored due to core aggregation prior to or during PVP coating. Niculaes et al. reported that small iron oxide nanocube clusters (dimers and trimers) increased specific absorption rate (SAR) values, while centrosymmetric clusters having more than four cores led to lower SAR values.^28^ Moreover, 1D (chain) arrangement IONPs also increased heating ability due to the dipolar interaction effect.^28^, ^29^ Overall, the multicored sIONPs did not significantly affect the heating ability of sIONPs, which will be discussed later (see Section 2.2.3).

The porous structure in the silica shell was observed in TEM images (Figure 1b; and Figure S4, a high‐resolution TEM image, Supporting Information). We hypothesized that the porosity in silica was due to the addition of PVP. Direct comparison of the silica coating on EMG308 without PVP was impossible due to our inability to coat EMG308 with silica in the absence of PVP. Instead, a comparison was made with pure silica nanoparticles (SiNPs) synthesized with and without PVP. Similar to the sIONPs, SiNPs synthesized with PVP were less electrically dense and revealed a porous structure with TEM (Figure 1c), while SiNPs without PVP were more electrically dense and solid. Although SiNPs prepared by the Stöber method are sometimes claimed as microporous,^30^ incorporating molecules into the silica matrix ensures larger pore formation.^31^ Macromolecules, such as tannic acid have been reported as a template for large mesopores.^32^ Small molecules such as glycerol were reported as templates for microporous silica.^31^ Fujita et al. reported the ability to create hollow silica nanoparticles using hydrophobic amines by using confined globular PVP composites as templates.^33^ PVP was also used as a contemplate with mesoporous templates, such as CTAB and triblock copolymer to assist formation of hollow mesoporous silica spheres^34^ and rod‐shaped mesoporous silica^35^ due to the association with micelle structures. Although PVP has been used to assist silica coating of a variety of nanoparticles,^22^, ^26^ it has not to our knowledge been previously reported as a porogen in silica.

sIONPs with and without surface modification were analyzed by nitrogen adsorption analysis. Both isotherms showed hysteresis at high relative pressure, indicating interparticle spaces due to the rough surface (Figure 1d). The unmodified sIONPs showed a steep increase of adsorption at very low relative pressure (0.0037), and hysteresis at low relative pressure indicates microporosity. As a result, the pore size distribution calculated by Barrett, Joyner, and Halenda method showed micropores ≤2 nm within the silica shell of the unmodified sIONPs. Unfortunately, pore size distribution below 2 nm could not be obtained due to instrumental limitations. Moreover, surface‐modified PEG blocked the pores, so no pore structure could be detected on modified sIONPs.

#### 2.1.2 Scale‐Up

One notable advantage of sIONPs is their simple synthesis method which allows scaled‐up production to gram quantity in the lab. This requires the use of a probe sonicator (Q500, Qsonica, rather than bath sonicator as reported previously^22^) and an overhead mechanical stirrer (rather than a magnetic stirrer^22^). Unlike a bath sonicator, which is mild and does not have uniform power across the bath, the probe sonicator is at least 100 times more powerful and the amplitude is controllable and tunable.^36^ By switching the probe diameter, we were able to sonicate solution volumes ranging from 1 mL to several liters. A magnetic stirrer was found to be inadequate for producing msIONPs in a reaction solution above 300 mL as this resulted in polydispersity of resulting nanoparticles and IONP cores shifting from the center to the edge of the silica coating. With an overhead magnetic stirrer (OS20‐s, Waverly), sIONPs can be produced in a 4L reaction vessel. The scaled‐up sIONP synthesis protocol currently yields above 20 g (or 1.4 g Fe) sIONP/batch, which is more than 80 times the original msIONP synthesis yield (0.017 g Fe mL^−1^).^22^ We believe sIONPs could be further scaled up in a larger reaction vessel in the near future with our industry collaborator.^37^

### 2.2 sIONP Characterization

#### 2.2.1 Surface Characterization

Besides direct observation of silica shells in TEM images, XPS confirmed the silica coating on the EMG308 in the presence of Si 2p binding energy and the disappearance of Fe binding peaks (Figure S5, Supporting Information). The N1s peak observed is due to the impurity of residue ammonium (catalyst) used in the silica coating reaction. The bare and modified sIONPs show no difference in morphology (data not shown), while IR revealed organic ligands on the surface of modified sIONPs (Figure S6, Supporting Information). Due to the low ratio of organic material to the bulk inorganic nanoparticle, the IR peaks were shallow yet still informative. The peaks arising in the modified sIONPs at 3450, 2880, 1460, and 1340 cm^−1^ were assigned to O—H stretching, C—H stretching, C—H bending from PEG, and methyl groups on the surface, respectively.^38^ The bands due to C—O, C—O—C, and C—O—H modes overlapped with the SiO_2_ peak in the region of 1300–900 cm^−1^ and could not be identified.^38^, ^39^ The small peaks in the region of 2200 were due to the CO_2_ in the air.

TGA showed similar weight percentage losses of both bare and modified sIONPs (Figure S7, Supporting Information). The major (≈12%) weight loss observed is caused by the imprinted PVP, which helps creates micropores in the silica shell.^40^ PVP is considered a low‐toxicity polymer^41^ and has been added to CPA cocktails to alter the CCR;^7^ therefore, the PVP in sIONPs was not a toxicity concern in our study. Moreover, the toxicity studies (see Section 2.3.1) indicate no impact in toxicity from imprinted PVP in sIONPs. The first weight loss (between 30 and 200 °C) was due to water evaporation. The second weight loss (between 400 and 500 °C) was due to the decomposition of organics, mostly PVP.^40^ Although the weight loss from PEG and TMS decomposition was negligible compared to the weight loss due to imprinted PVP, the 1st derivative thermo‐decomposition temperature of the modified sIONPs shifted higher (423–454 °C) than the bare sIONPs, indicating the covalent bonding of organics on the sIONPs since the thermo‐decomposition temperature of nanoparticle‐bonded polymers is higher than free ones.^42^ Moreover, the decrease in sIONP zeta potential from −72 ± 4 to −40.29 ± 8 mV after modification also suggests attachment of PEG and methyl groups that partially neutralized the negatively charged silanol groups on the surface.

#### 2.2.2 Colloidal Stability in CPAs

Aggregation of IONPs in CPAs leads to reduced heating,^21^ heterogenous distribution of IONPs and, as we will show, blocking of the vasculature. Colloidal stability of IONPs in CPAs is more challenging than in other biological media (i.e., saline or phosphate buffered saline (PBS)) due to high viscosity and high concentrations of salt, sugars, and organics. For instance, IONPs that are stable in PBS such as PBG300, a PEGlyated IONP from Ferrotec, are not stable in CPAs (data not shown). We presume this is due to the PEG being adsorbed instead of covalently bound to the IONPs.

**Figure 2:**
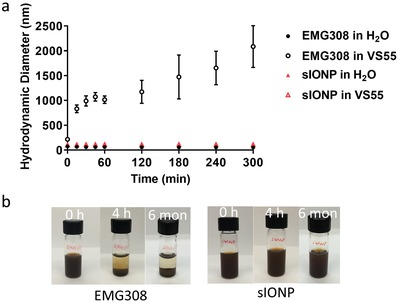
Colloidal stability of EMG308 and sIONPs. a) Hydrodynamic diameters of EMG308 and sIONPs, each measured using DLS in H_2_O and VS55 in the first 5 h following synthesis of the colloid. EMG308 aggregates in VS55, while sIONPs are stable in VS55. b) Photos of EMG308 and sIONP in VS55 for 0 h, 4 h, and 6 months. EMG308 completely crashes out from VS55 within 4 h, while sIONPs remain suspended in VS55 for at least 6 months.

sIONPs and EMG308 were dispersed in VS55 and monitored for colloidal stability. Although we previously observed EMG308 crash out from VS55 within several hours,[qv: 16b] we now show EMG308 aggregation in VS55 by DLS measurements over time (**Figure** **2**a). Using measured viscosity (3.26 cP) and refractive index (1.379) of VS55 at 23 °C (method described in the Experimental Section), we were able to calculate the hydrodynamic diameters of sIONPs in VS55 and found them to be identical to the one measured in water (104 nm in H_2_O and 106 nm in VS55). Interestingly, the aggregation started immediately after dispersing EMG308 in VS55. Within 30 min, the average hydrodynamic diameter of EMG308 aggregates grew to over 1 µm, and the polydispersity of the aggregates kept increasing. EMG308 was visually confirmed to crash out from VS55 in the 4th h (Figure 2b). Moreover, the aggregated EMG308 remains stably aggregated even after intensive sonication (data not shown). On the other hand, sIONPs, due to their surface modification, were stable in VS55 for at least 6 months at room temperature (Figure 2b).

#### 2.2.3 Heating Properties

**Figure 3:**
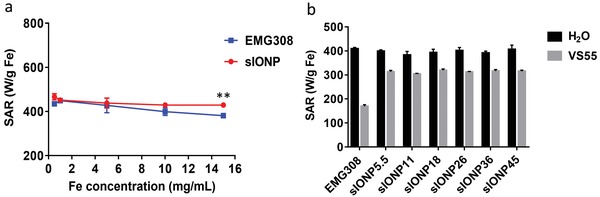
Heating performance, measured as SAR per gram Fe (at 360 kHz, 20 kA m^−1^), of a) EMG308 and sIONP water suspensions versus increasing concentrations, **p < 0.01. b) EMG308 and sIONPs with different shell thicknesses in water and in VS55. Data of EMG308 in VS55 were acquired when EMG308 was in suspension following vortexing. When the EMG308 crashed out of the solution, SAR was negligible.

Heating capability of IONPs is an essential factor for nanowarming applications. **Figure** **3**a shows that sIONP specific absorption rates of unit iron weight (SAR_Fe_) were constant at different Fe concentrations in water. In the same conditions, however, EMG308 showed a significant decrease of heating ability as Fe concentrations increased and interparticle interference, which is known to influence locally induced magnetic fields, became more intensive.^21^ It is likely that the silica coating acts as a steric spacer between the IONP cores, making sIONPs less affected by interparticle interactions.^43^ This silica steric buffer, then, is an important advantage to maintaining high heating with sIONP at high (>10 mg Fe mL^−1^) concentrations during nanowarming.

Direct heating comparisons for IONPs described in the literature are usually carried out in water. However, for nanowarming applications, it is vital that heating capability be assessed within CPAs. For instance, when changing the carrier solution from water to VS55, the SAR of EMG308 was reduced by more than 50% due to aggregation (Figure 3b). Moreover, no heating was detected once EMG308 crashed out of the solution. Although the heating rates of the sIONPs in VS55 were higher than they were in water, due to the lower specific heat capacity of VS55,^44^ the actual SAR in VS55 is lower than in water. Nevertheless, the SARs of sIONPs with various silica shell thickness held constant in both VS55 and water. The SAR of EMG308 and sIONPs with different silica shell thicknesses in water and in VS55 was obtained by measuring 1 mg Fe mL^−1^ samples. At this low Fe concentration, the interparticle interference was negligible.

One important question is whether the nanoparticles themselves change the thermal properties, or otherwise influence nucleation and/or crystallization in CPA solutions. Previous work in EMG308, the core IONP used in our sIONP formulation, shows negligible impact on the thermal properties of VS55 during cooling and rewarming.[qv: 16a,45] Indeed, one study even showed that EMG308 suppresses nucleation and stabilizes the glassy state.^45^ However, other studies suggest that certain surface formulations can promote devitrification in VS55.^46^ While we have seen no evidence to suggest relevant changes to thermal conductivity, vitrification and devitrification behavior of VS55 with sIONPs, ongoing work in our lab and that of our collaborator(s) is expected to answer this.

#### 2.2.4 Magnetic Properties

**Figure 4:**
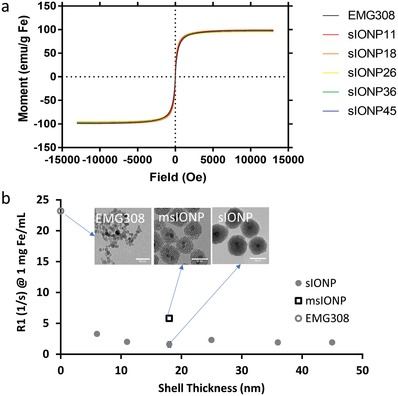
a) Hysteresis loop of sIONPs with various shell thicknesses at room temperature. The influence of silica shell to EMG308 magnetic saturation momentum is negligible. b) The longitudinal relaxation rate constant (R_1_) at 9.4T of EMG308, msIONPs, and sIONPs with various shell thicknesses at 1 mg Fe mL^−1^. For comparison, the R_1_ at 1 mg Fe mL^−1^is shown for EMG308 (sIONP with shell thickness = 0 nm) and msIONP.[qv: 23b] Error bars indicate the standard deviation across the sample and are not visible because they are smaller than the circular marker shown. The data show that silica coating hinders the water access to the core and reduces R_1_.

Because heating arises from the magnetic properties of sIONPs, we undertook further characterization by magnetometry. **Figure** **4**a shows the hysteresis loops of powdered EMG308 and sIONPs with various shell thicknesses. Measurements were performed at room temperature. All IONPs showed negligible DC hysteresis, indicating that they are all superparamagnetic at room temperature. The magnetization remains constant,^47^ and the negligible variations between the samples were likely due to the measurement errors instead of silica coating.

MRI measurement of relaxation time constants (i.e., the longitudinal and transverse relaxation time, T_1_ and T_2_, respectively) are valuable for evaluating sIONP distributions within an organ.[qv: 16b,23b] Low concentrations (<0.01 mg Fe mL^−1^) of IONPs, such as those observed after organ washout, can be measured using a T_2_‐based MRI method.[qv: 23b] Higher concentrations of IONPs (0.01–3 mg Fe mL^−1^), such as those present in the microvasculature of an IONP‐loaded organ, can be measured using a T_1_‐based MRI method with ultrashort echo time, such as sweep imaging with Fourier transformation (SWIFT).^48^ Initial measurements were focused on the longitudinal relaxation rate constant R_1_ (= 1/T_1_) of a range of silica shell thicknesses at 1 mg Fe mL^−1^ in 1% agarose (Figure 4b). The R_1_ values for all of the sIONPs are lower than those for uncoated EMG308 and msIONPs.[qv: 23b] The decrease in R_1_ can be attributed to reduced water accessibility to the core. The R_1_ is observed to barely change between 6 and 45 nm shell thicknesses. This is a deviation from Pinho et al., who reported a dramatic decrease in R_1_ when silica shell thickness increased from 7.6 to 42.7 nm.^47^ We hypothesize that the discrepancy in response is best attributed to further inhibition of water diffusion from the PEG coating. A similar impact on decreased pore accessibility due to the presence of PEG was shown in the nitrogen adsorption data (see Section 2.1.1). Additionally, the relaxation rate as a function of concentration or relaxivity (r_1_ and r_2_) and the heating rate (volumetric specific absorption rate, SAR*_v_*) were measured on sIONPs with a 16 nm silica shell suspended in 1% agarose and VS55. The 1% agarose and VS55 were used to mimic the nanowarming environment. The reported r_1_ is lower than previously published r_1_ values for msIONPs and EMG308.[qv: 22,23b] A good correlation between R_1_ and heat production (SARv) was observed (Figure S8, Supporting Information). Furthermore, the lower R_1_ allows for the ability to image the higher sIONP concentrations necessary for nanowarming.

### 2.3 Biology Interaction and Applications

#### 2.3.1 Cytotoxicity

**Figure 5:**
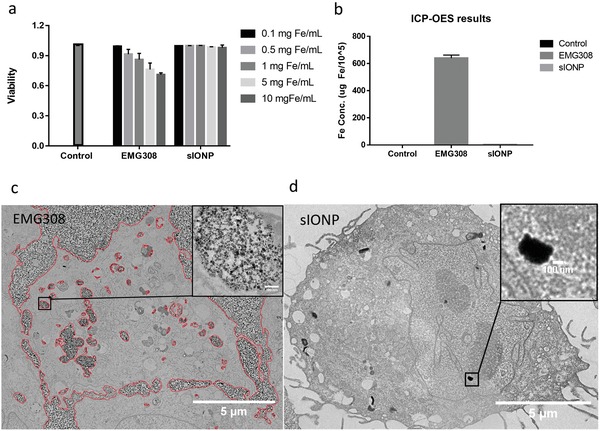
a) Viability of HDFs after incubation with EMG308 and sIONPs with increasing concentrations. EMG308 showed toxicity to HDFs when the incubation concentration was above 0.1 mg Fe mL^−1^. sIONPs did not reduce HDF viability at all the tested concentrations. b) Fe quantification of HDFs exposed to EMG308 and sIONPs for 24 h by ICP‐OES. c) TEM images of EMG308 association with HDFs, with red circles indicating EMG308. The zoom‐in image on the right shows EMG308 up taken by the cell. d) TEM image of a cell exposed to sIONPs. The zoom‐in image on the right shows a dark granule. ICP‐OES and TEM both indicate EMG308 intensively associate with HDFs, while sIONPs showed minimal cellular association.

Although the expectation is that the majority of sIONPs will be washed out of the biological samples, complete removal of sIONPs is impossible and the toxicity of residual sIONPs is a concern. Thus, the toxicity of EMG308 and sIONPs were tested in a wide range of Fe concentrations (0.1–10 mg Fe mL^−1^) on human dermal fibroblasts (HDFs). EMG308 reduced cell viability starting from 0.5 mg Fe mL^−1^ as shown in **Figure** **5**. The toxicity of EMG308 might be from a commercially added anionic surfactant and intensive uptake and association of EMG308 with the cells. In contrast, there was no statistically significant decline of cellular viability even at the highest Fe concentration tested for sIONPs (Figure 5a). This is consistent with silica being biocompatible and the PEG and the additional methyl group leading to little or no cellular interaction as reported for similar structures in the literature.^22^ In summary, our biocompatibility tests demonstrated that sIONPs are nontoxic at least up to exposure concentrations as high as 10 mg Fe mL^−1^ for 24 h in HDF cells.

#### 2.3.2 Cellular Interaction

IONPs that show minimal cellular interactions are preferred in nanowarming to reduce residual IONPs left within the organ after rewarming. Therefore, the associations between IONPs and cells were evaluated. sIONPs and EMG308 were incubated with HDFs for 24 h at 37 °C. After washing with Hank's balanced salt solution (HBSS) buffer 5 times to remove the unassociated IONPs, the cells were collected by centrifugation and then embedded in resin for TEM imaging. The presence of EMG308 could be observed in the cell pellet by a dark brown coloration. The pellet from cell exposure to sIONPs had a slightly yellow color compared to the control, which was white. Ultrastructure images acquired with TEM (Figure 5c) show rare uptake and thus little sIONP association with cells. sIONPs were observed within the void spaces between the cells but did not attach to the cell membranes. High uptake and attachment to the cell membrane was observed in cells incubated with EMG308. This observation is consistent with cellular association comparisons made between EMG308 and msIONPs on LNCaP cells.^22^ The cellular association between EMG308 and sIONPs was quantified by ICP‐OES (Figure 5b). The small amount of iron detected with the sIONP‐incubated cells (300 times less than EMG308) is consistent with TEM observations. The low cellular interaction of sIONPs showed promise that they will be easily washed out of organs after nanowarming.

#### 2.3.3 Nanowarming of Cells

**Figure 6:**
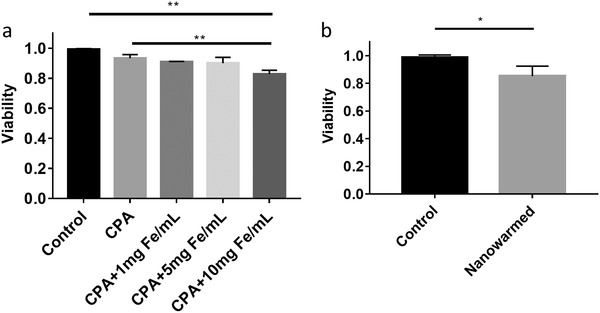
Using sIONPs for HDF nanowarming. a) Toxicity of sIONP in CPA at 4 °C. The exposure of CPA and sIONP showed some toxicity to the cells. b) Cell viability after nanowarming is comparable with our previous results using msIONPs.[qv: 16b] *p < 0.05, **p < 0.01.

Initial nanowarming demonstrations with msIONPs were first demonstrated in a cell system.[qv: 16b] We repeated the experiment with sIONPs to show equivalency. Cellular systems are advantageous as initial nanowarming tests because the toxicity of each nanowarming component (CPA, IONPs, cooling, and heating) can be separately evaluated. Therefore, initial measurements assessed the toxicity of the CPA with increasing concentrations of sIONP exposures (**Figure** **6**a).[qv: 16b] The CPA was loaded by exposing HDFs with increasing CPA concentration solutions and removed by exposing HDFs with lower CPA concentration solutions on ice (see the Experimental Section). The sIONPs were introduced to the cells with the highest concentration of CPA and removed during the CPA unloading steps. The viability of cells that exposed to CPA and 10 mg Fe mL^−1^ sIONPs showed a slight decline from the negative control samples in culture medium (*p* < 0.01), while lower concentrations of sIONPs did not significantly affect the cell viability. Then, cell viability recovered from vitrification (cooling at ≈7 °C min^−1^) and nanowarming (≈130 °C min^−1^) was tested. A recovery viability of 85.3% was achieved with sIONP nanowarming (Figure 6b), which was comparable to the previous reported results using msIONPs (83.6%).[qv: 16b]

#### 2.3.4 Loading and Washout of IONPs from Rat Kidneys

In order for nanowarming of organs to work, IONPs will need to be loaded prior to cryopreservation and eventually washed out after rewarming. Here we provide a first demonstration of the ability to load and washout IONPs in VS55 from an organ. First, rat kidneys were preloaded with VS55 in a stepwise manner (Euro‐Collins, 18.7%, 25%, 50%, 75%, 100% VS55)[qv: 16b] at a constant flow rate of 3 mL min^−1^. Then EMG308 or sIONPs (10 mg Fe mL^−1^) in VS55 suspensions were perfused into the rat kidney through the kidney infrarenal aorta and then washed out with stepwise decreasing VS55 solutions.

**Figure 7:**
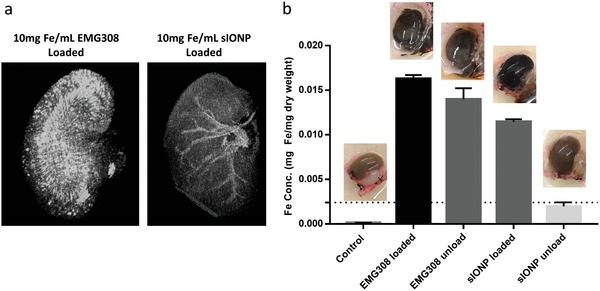
a) Micro‐CT 3D projections for IONP‐loaded kidneys corroborate aggregation in kidneys for uncoated EMG308 (left) compared to sIONPs (right). b) ICP‐OES results of control, IONP‐loaded and IONP‐washed‐out kidneys and the corresponding photos. The ICP‐OES results indicate that stable sIONPs can be washed out more effectively than uncoated IONPs, which aggregate during CPA perfusion. Error bars are standard deviation.

The kidney, once fully loaded with sIONPs, was imaged by µCT, showing distribution in the major vessels and capillaries. EMG308 agglomeration in the vasculature was indicated by the intensive contrast all around the kidney (**Figure** **7**a). This agglomeration in the vasculature was also indicated by the high pressure (≈250 mm Hg) needed to overcome blockage of the vasculature, especially compared with the lower washout pressure (≈100 mm Hg) of sIONP‐loaded kidneys (Figure S9, Supporting Information). Moreover, the EMG308‐loaded kidneys showed higher iron content (0.0163 mg Fe mg^−1^ dry weight) than sIONP‐loaded kidneys (0.0115 mg Fe mg^−1^ dry weight) by ICP‐OES (Figure 7b). The washed‐out kidneys were analyzed by MRI and ICP‐OES to assess residual iron. In the case of EMG308, loaded kidneys were visibly darker than negative controls (i.e., unloaded control kidneys), and kidneys after washout were still visibly dark (Figure 7b). According to ICP‐OES results, 86% of the loaded EMG308 remained in kidneys after washout and produced artifacts within the MRI due to the high iron concentration (Figure S10, Supporting Information). In contrast, the sIONP washed‐out kidney was visually similar to the negative control (Figure 7a). However, T_2_‐weighted MRI indicated the presence of sIONP residue post washout (Figure S10, Supporting Information). The ICP‐OES results show 0.0020 mg Fe mg^−1^ kidney dry weight, which is within the detection limits of T_2_‐weighted MRI based on our r_2_ measurements (Figure S8, Supporting Information). The iron concentration remaining in the sIONP‐loaded kidney is an order of magnitude lower than that remaining in the EMG308‐washout kidney (0.0140 mg Fe mg^−1^ dry weight). Although sIONPs cannot be completely removed from the kidney, in vivo studies of mice i.v. tail vein injected with msIONPs showed slightly higher concentrations 24 h postinjection (0.0024 mg Fe mg^−1^ kidney dry weight), which was tolerated without adverse reaction for over a month.[qv: 23a] From these observations, we hypothesize that the residual sIONPs within the kidney are unlikely to induce toxicity.

## 3 Conclusion

With the aim of scaling nanowarming technology to organs, a new silica‐coated iron oxide nanoparticle, sIONP, was produced and tested against existing IONPs, especially EMG308 and msIONPs, which were used for the first physical and biological demonstration of nanowarming, respectively. Here we show that sIONP synthesis is simple, fast, and less labor‐intensive than msIONP synthesis and eliminates the need to use toxic surfactants. As a novel nanoparticle, the morphology and surface of sIONPs were carefully characterized. We show that sIONPs' heating and magnetic properties remain constant regardless of shell thickness. Further, the sIONPs are shown to be biocompatible with minimal cellular interaction with HDFs. Finally, sIONPs and EMG308 in VS55 were perfused into rat kidneys and analyzed by µCT. The sIONPs distribute throughout the vasculature, while EMG308 showed severe agglomeration followed by perfusion pressure changes that indicate blockage. Further, more than 90% of loaded sIONPs were removed during CPA washout, while the majority of EMG308 remained stuck in the vasculature. ICP‐OES showed that the remaining amount of sIONPs was below the known amount tolerated in kidneys after in vivo administration in a separate longitudinal study.

## 4 Experimental Section

*Chemicals*: TEOS, and polyvinylpyrrolidone (PVP‐10, average molecular weight 10000), Chlorotrimethylsilane (TMS, > 99%) were purchased from Sigma‐Aldrich. 2‐[methoxy(polyethyleneoxy)‐propyl]9‐12‐trimethoxysilane (PEG‐silane) was obtained from Gelest, Inc. Ethanol (99%) was purchased from Pharmco‐Aaaper. EMG308 Ferrofluid was purchased from Ferrotec. Ammonium hydroxide (NH_4_OH, 28%) was obtained from Avantor Performance Materials.

*sIONP Synthesis*: A prototype small‐scale synthesis was conducted in a 150 mL Erlenmeyer flask using a magnetic stir bar for mixing. 0.6 g PVP10 was dissolved in water by sonication, then probe sonicated for 5 min. After adding 18 mg Fe stock EMG308 to the PVP solution and ensuring that the total amount of water was 5.4 mL, the mixture was probe sonicated for 10 min. The EMG308 PVP solution was then added to a flask with 40 mL ethanol, and probe sonication was continued for another 10 min. 2 mL ammonia was added to the reaction while stirring. 0.5–8 mL TEOS was added to the solution while stirring at room temperature. 0.25 mL PEG saline was added after 1 h. 0.0375 mL TMS was added after another half an hour. The reaction continued overnight to allow complete condensation. The resulting sIONPs were collected by ultracentrifugation at 30 000 rpm for 15 min and washed with ethanol, ethanol/water mixtures, and water for several cycles to remove unreacted reagents. The purified sIONPs were then redispersed in water and filtered to remove micrometer‐sized impurities or aggregates.

*sIONP Scale‐Up*: The scale‐up synthesis was done in a 4L reaction vessel with an overhead mechanical stirrer used for mixing. For sIONPs with 18 nm silica shell, 48 g PVP10 was dissolved in water, and 1.440 g Fe EMG308 was added to preprobe sonicated PVP10 solution (water volume is 432 mL) and probe sonicated (Q500, Qsonica) for 45 min. Then the mixture was added to 3.2 L ethanol and probe sonicated for another 45 min while stirring. 160 mL ammonia was added to the 4L reaction vessel (LG‐8082‐104, Wilmad‐LabGlass) while stirring by an overhead mechanical stirrer (OS20‐S Waverly). 80 mL of TEOS was added afterward while stirring. 20 mL PEG silane was added to the mixture after 1 h and stirring continued. 3 mL TMS was added after another 30 min. After the reaction, the reaction solution was concentrated by rotary evaporator, and sIONPs were collected and purified via repeat centrifugation.

*sIONP Characterizations*: sIONPs were characterized by DLS and Zeta potential measurements on a Brookhaven Zeta PALS instrument (Brookhaven Instruments Corporation) with a 635 nm diode laser at 15 mW of power. TEM was performed on a Tecnai T12 transmission electron microscope (FEI, OR) operating at 120 kV. ICP‐OES quantitation of iron was performed on a Thermo Scientific iCAP 6500 dual‐view ICP‐OES with 1150 W power. XPS was measured on a PHI5000VersaProbeIII. IR was measured on a Thermo Scientific Nicolet iS 50Ft‐IR using attenuated total reflection technique. Nitrogen adsorption–desorption measurements were performed on a Micromeritics ASAP 2020 surface area and porosity analyzer. The samples were degassed at 120 °C for 6 h prior to physisorption analyses. TGA were performed on a NETZSCH STA 409 PC Luxx system coupled with a NESLAB RTE‐101 bath circulator. The sample chamber was purged with high purity nitrogen (20 mL min^−1^) for 4 h prior to the analysis. TGA was performed in nitrogen using a ramp rate of 10 °C min^−1^ from room temperature to 900 °C. A MicroMag Vibrating Sample Magnetometer (Princeton Measurements Corporation) was used to measure the hysteresis loop of IONPs in a powder form at room temperature. The viscosity of VS55 was measured by an AR‐G2 rheometer at 23 °C. The reactive index was measured followed the procedure reported by An.^49^

*sIONP Heating Experiment Setup*: 1 mL of each sample (EMG308 or sIONPs in water or in VS55) was placed in a 1.75 mL Eppendorf tube and heated in 1 kW Hotshot inductive heating systems with 2.75‐turn, water‐cooled copper coil (Ameritherm Inc., Scottsville, NY) at 360 kHz and 20 kA m^−1^. The SAR was calculated based on linear regression of the first 30 s heating data (2–3 s lag time), with the heat generated from the container, water, or VS55 were subtracted.[qv: 16a]

*MRI Measurements*: MRI measurements including images and relaxation values, were performed with a 9.4T‐31 cm bore MRI scanner (Agilent Technologies, Santa Clara, CA). All images were acquired with a volume transmit/receive coil having an inner diameter of 3 cm (Agilent Technologies, Santa Clara, CA). Relaxation rate measurement of IONPs was performed in 1% agarose, following previously established protocols.[qv: 23b]

A multislice T_2_‐weighted spin echo sequence was used to measure T_2_‐weighted images and R_2_ maps. Each 2D image was acquired with a repetition time (TR) of 2.4 s, echo time (TE) of 12 ms, acquisition bandwidth of 50 kHz, and acquisition time of 2.56, a slice thickness of 5 mm, and a resolution of 417 × 417 µm. For R_2_ determination, six time points were acquired with TE spaced exponentially between 12 and 800 ms. All 2D images were reconstructed using VnmrJ version 3.2.

3D T_1_‐weighted images and R_1_ maps were acquired using a Look‐Locker method together with a MultiBand (MB)‐SWIFT sequence for readout.[qv: 23c,48] The MB‐SWIFT flip angle was 1°, acquisition delay ≈2 µs, acquisition bandwidth = 384 kHz, TR = 1.2 ms, gaps = 2, *N*_spiral_ = 32, and *N*_v_ = 4096, voxel resolution = 195 × 195 × 781 µm, and total acquisition time ≈7 min.^24^, ^29^ The field‐of‐view was 50 × 50 × 200 mm^3^ with image matrix size = 256 × 256 × 256 × 64 (x,y,z,t). 64 time points were spaced linearly from 39.8 to 4596 ms. MB‐SWIFT images were reconstructed using an in‐house program written in MATLAB (2012b).^50^

For each voxel, the time points were fit to the exponential curve using a three‐variable fit.[qv: 23c] The relaxation rate constant (R_1_ or R_2_) was determined with least‐squares fitting. The region of interest assessed for each tube was approximated as a cuboid with dimensions 2.73 × 2.73 × 25 mm^3^. The relaxivity (r_1_ or r_2_) was determined by performing a linear least‐squares fitting for the relaxation rates as a function of iron concentration.

*Microcomputer Tomography (microCT) Measurements*: microCT measurements were acquired in a Nikon XT H225 (Melville, NY). The images were reconstructed using 3D CT pro, Nikon Metrology, imported as unsigned 16‐bit float images. The reconstruction was corrected for beam hardening and denoising (75% Hanning filter). 3D maximum image projections were created in ImageJ.

*Cell Experiments*: Human dermal fibroblasts (HDF, ATCC) were cultured in Dulbecco's modified Eagle media (Gibco, life technologies) that contained 10% fetal bovine serum (Gibco, life technologies) and 1% penicillin streptomycin (Sigma) at 37 °C under 5% CO_2_. The nanoparticle toxicity test was conducted by incubating HDFs with various concentrations of EMG308 or sIONPs for 24 h, then evaluating the HDFs by Hoechst‐PI assay. The cellular association experiments were done in T‐75 flasks. HDFs were exposed to 1 mg Fe mL^−1^ EMG308 or sIONP for 24 h at 37 °C in an incubator. The cells were rinsed with HBSS five times to remove free IONPs. The cells were then collected and analyzed by TEM and digested for ICP‐OES. The CPA and sIONPs exposure toxicity experiments were done by stepwise loading and unloading of VS55 and sIONPs (loading steps: Euro‐Collins solution, 18.7% VS55, 25% VS55, 50% VS55, 75% VS55, 100% VS55 with 10 mg Fe mL^−1^ sIONP; removal steps: 50% VS55, 18.7% VS55, Euro‐Collins solution, cell culture media) in 3 min steps. The cryopreserved HDFs were cultured in an individually cut 96 well and placed in a 1 mL cryovial for cooling and rewarming. The cooling was conducted in a home‐made multilayer cooler by liquid nitrogen vapor (cooling rate at ≈7 °C min^−1^) and rewarmed in 1 kW RF coil at 360 kHz and 20 kA m^−1^ (rewarming rate at ≈130 °C min^−1^). A Hoechst‐PI assay was used to evaluate cell viability.

*Kidney Experiments*: All animal experiments were approved by the University of Minnesota Institutional Animal Care and Use Committee (IACUC). Male Lewis rats 2–3 months old, weighing from 200 to 250 g, had general anesthesia induced with 4% of isoflurane and 1 L min^−1^ oxygen and maintained with 1.5% of isoflurane and 0.9 L min^−1^ oxygen. The adequacy of anesthesia was confirmed by toe pinch reflex. The hair of the abdominal area was shaved and abdominal skin disinfected by using 70% Ethanol solution. A long midline incision was made, and the abdominal organs retracted to the left side of the abdominal cavity. The aorta and Inferior vena cava (IVC) were mobilized from distal bifurcation up to the superior mesenteric artery (SMA) level. All the branches from IVC and Aorta were ligated by 6.0 silk ties. Care was taken to protect the left renal artery and vein. 3.0 silk loose ties were placed below the SMA (proximal aorta) and above the iliac bifurcation (distal aorta). 500 IU Heparin was given via the dorsal penile vein. 2 min later loose tie on the tied distal aorta. 20G IV catheter was inserted into the distal aorta and secured with 3.0 silk tie and connected to 30 mL syringe with cold Euro‐Collins solution. The proximal aorta was tied and the IVC was transected below the renal vein. The left kidney was perfused with 30 mL cold Euro‐Collins solution. Once flushed, the left kidney was immediately excised and transferred in Euro‐Collins solution on ice and connected to a perfusion setup at a constant flow rate of 3 mL min^−1^. The perfusion pressure was monitored and recorded during the perfusion. VS55 was loaded in a stepwise manner: Euro‐Collins, 18.7% VS55, 25% VS55, 50% VS55, 75% VS55, 100% VS55 at 4 °C with each step lasting 15 min. After the VS55 loading, a 10 mg Fe mL^−1^ sIONP or freshly made EMG308 VS55 solution was loaded to the kidney at 1 mL min^−1^ for about 2–3 min or until the kidney turned to black. The resulting loaded kidneys were then sutured and imaged by MRI and µCT within 72 h of perfusion. After imaging, the kidneys were dried in a vacuum oven at 130 °C overnight for ICP‐OES measurements. Then 75% VS55, 50% VS55, 25% VS55, 18.7% VS55, Euro‐Collins were perfused for 15 min in each solution to remove IONPs and VS55. The kidneys after IONP washout were placed in Euro‐Collins solution for MRI imaging within 72 h of perfusion and then freeze dried for ICP‐OES measurements.

*ICP‐OES Sample Preparation*: IONP samples were digested in a 0.3 m ascorbic acid and 0.3 m HCl solution at 60 °C for 3 h. The biological samples were first dried and then ground to fine powders. The powder sample (≈45 mg) was predissolved in a mixture of 0.6 mL concentrated HNO_3_ and 0.3 mL H_2_O_2_ overnight. After that, the samples were sealed in a 6 mL microwave digestion vessel inside a 60 mL microwave digestion vessel with 10 mL H_2_O in the larger vessel. The microwave digestion was performed in a domestic microwave at 50% power for 3 min, cool down, pressure release, 50% power for 3 min, cool down. The digested solution was then diluted to 10 mL with 2% nitric acid. The resulting solution was then digested similar to the IONP samples in a 0.3 m ascorbic acid and 0.3 m HCl solution at 60 °C for 3 h.

*Statistical Analysis*: All the physical measurements and viability experiment were repeated at least three times. Statistical significance is indicated with asterisks: * *p* < 0.05; ** *p* < 0.01; *** *p* < 0.001 **** *p* < 0.0001. The error bars are standard deviations. The one‐way analysis of variance (ANOVA) with Tukey's multiple comparison tests (GraphPad Prism, GraphPad Software, Inc.) was performed on viability data. A two‐tailed *t*‐test was performed on the analysis of physical data.

## Conflict of Interest

The authors declare no conflict of interest.

## Supporting information

Supporting Information
