# Supplementary material for: Preparation of Scalable Silica‐Coated Iron Oxide Nanoparticles for Nanowarming
Source: Adv Sci (Weinh). 2020 Jan 7;7(4):1901624. doi: 10.1002/advs.201901624 (PMC7029634; doi:10.1002/advs.201901624)
Supplement: Supplementary file 1 — Supporting Information [file ADVS-7-1901624-s001.pdf]

Copyright WILEY-VCH Verlag GmbH & Co. KGaA, 69469 Weinheim, Germany, 2020.

ADVANCED SCIENCE
Open Access

Supporting Information

for *Adv. Sci.*, DOI: 10.1002/advs.201901624

Preparation of Scalable Silica-Coated Iron Oxide Nanoparticles for Nanowarming

*Zhe Gao, Hattie L. Ring, Anirudh Sharma, Baterdene Namsrai, Nam Tran, Erik B. Finger, Michael Garwood, Christy L. Haynes, and John C. Bischof**

Copyright WILEY-VCH Verlag GmbH & Co. KGaA, 69469 Weinheim, Germany, 2019.

Supporting Information

Preparation of Scalable Silica-Coated Iron Oxide Nanoparticles for Nanowarming

*Zhe Gao, Hattie L. Ring, Anirudh Sharma, Baterdene Namsrai, Nam Tran, Erik B. Finger, Mike Garwood, Christy L. Haynes, John C. Bischof**

Table S1 Summary of using electromagnetic heating for volumetric rewarming

Year	Heating Method	Settings	Rewarmed material	Results
1977-1979[12b, 12e, 12f]	Domestic microwave ovens	2450 MHz	dog kidneys	Success was non-repeatable
1990[11]	RF helical coil	20-30 MHz	30-120 mL CPA	Average heating rate was 20 °C min^{-1}100 W^{-1}100mL^{-1}However, large volume needs high power supply.
1999[12a]	Optimized electromagnetic heating	434 MHz	36-mm CPA sphere	Fast and uniform heating limited within small spheres.
2019[18]	Magnetic nanoparticle enhanced electromagnetic heating	Dynamically controlled field starting at 430 MHz.	20 mL CPA solution with IONPs	Physically demonstrated uniform heating >200 °C min^{-1}
Ongoing [17]	Electromagnetic and conductive hybrid heating	-	20 mL cell suspension	Viability increased to 82%

Table S2 Comparison of msIONP and sIONP synthesis

	Yield [g $batch^{-1}$]	Time [day]	Steps				
msIONP	0.035	5	Synthesis	Deoxy-genation	Hydrothermal treatment	CTAB removal	Centrifugation purification
sIONP	0.2-1.4	1-2	Synthesis		Centrifugation purification		

(Red signifies elevated temperature.)

Table S3 Consumption of IONPs in organs

	Rat			Rabbit			Human		
	Liver	Kidney	Heart	Liver	Kidney	Heart	Liver	Kidney	Heart
Organ mass [g]	12	4	2	84	13	5	1561	137	308
Vasculature mass [g or mL]	2.04	1.02	1	14.28	3.32	2.5	165.37	35	154
Estimated total Fe required [mg]*	90.8	70.4	70	335.6	116.4	100	3357.4	750	3130

(*Fe required amount is calculated at 10 mg Fe mL^{-1} in the vasculature. In reality, much more Fe is required for the perfusion loop consumption, complete perfusion, and in surrounding solution. We would expect 1-2 mL for the loop consumption and an extra 30-60 s to allow complete perfusion at 3mL min^{-1}. The Fe concentration in the surrounding solution is estimated as the same Fe concentration as in Fe perfused in unit organ volume. The goal is for the surrounding solution heating rate to match the heating rate of sIONP-loaded organs. The surrounding solution volume depends on the container. The surrounding solution should ensure the complete submergence of the organ, so we expect the surrounding solution's volume to be the same as the organ volume.)

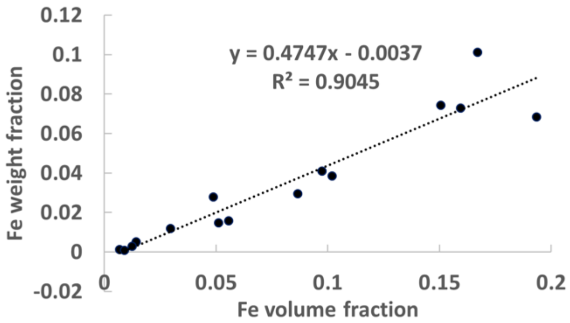

Figure S1. Correlation of Fe volume fraction and weight fraction.

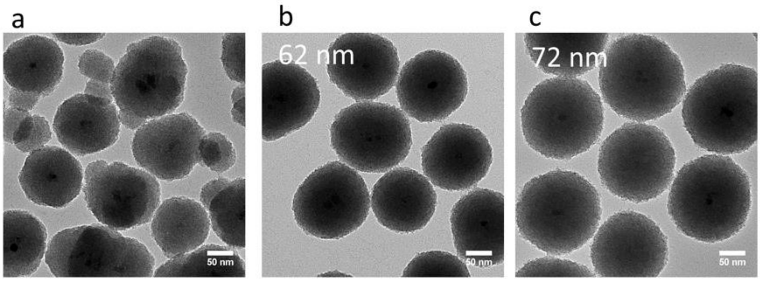

Figure S2. a) Excess TEOS during synthesis will cause formation of free silica nanoparticles. b,c) It is possible to increase the shell thickness by multistep addition of TEOS.

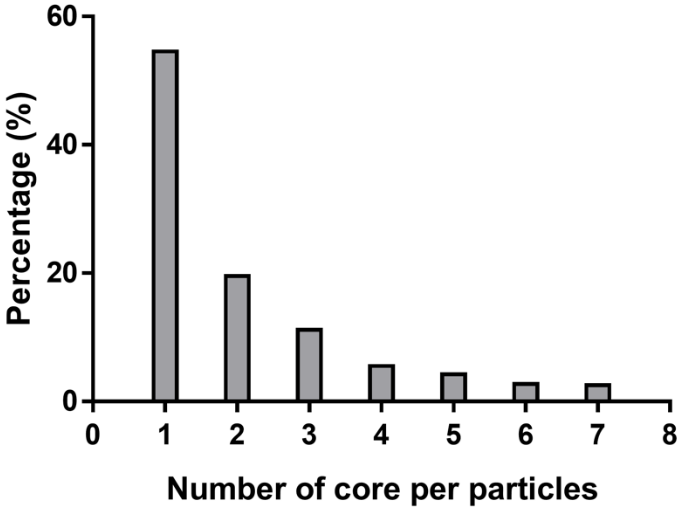

Figure S3. Number of cores per particles (N=565).

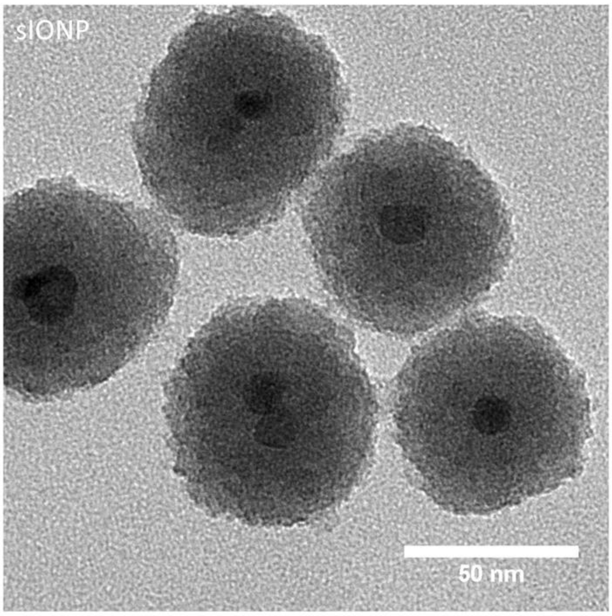

Figure S4. High resolution TEM image of sIONPs shows the porosity of the silica shells.

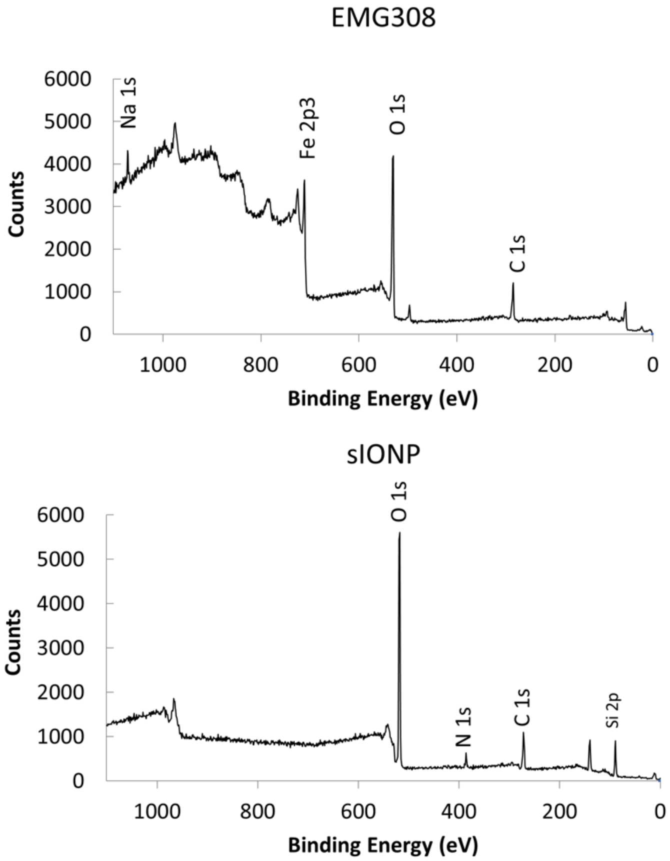

Figure S5. XPS survey data of EMG308 and sIONPs.

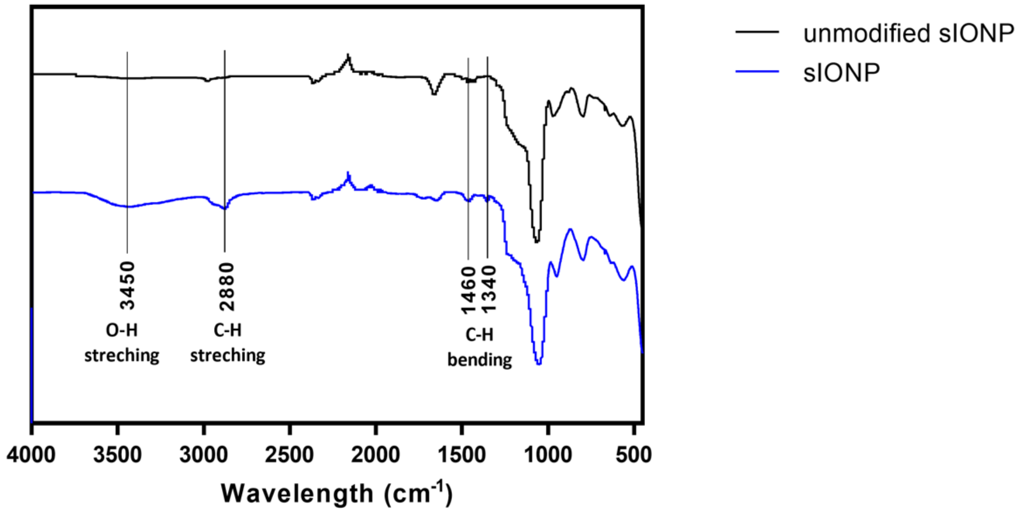

Figure S6. IR data of sIONPs with and without surface modification.

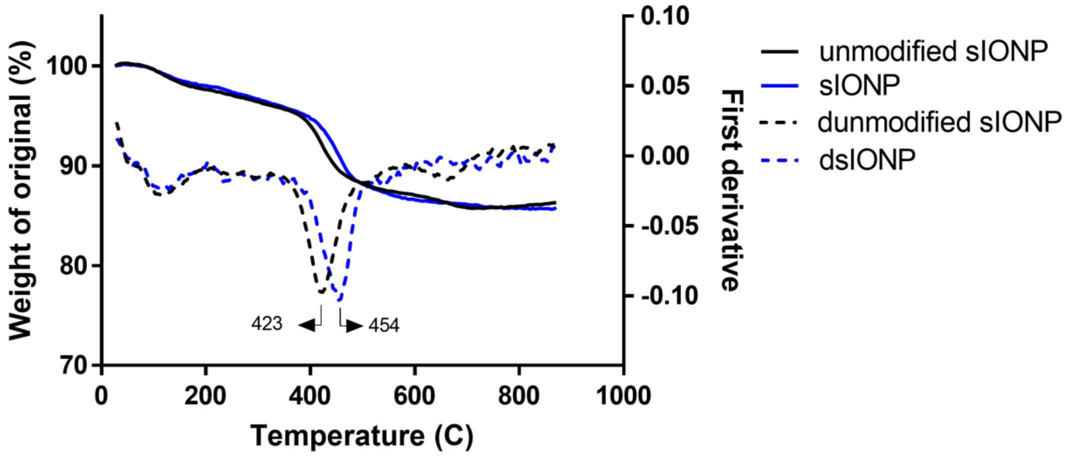

Figure S7. TGA data of sIONPs with and without surface modification. The major weight loss is due to the imprinted PVP. The 1st derivative peak of thermal decomposition temperature is higher in the modified sample due to the covalently bonded surface ligands.

Parameter	Value
r_1	0.317 $(mg_{Fe}/mL)^{-1}s^{-1}$<br>0.018 $mM_{Fe}^{-1}s^{-1}$
r_2	4574 $(mg_{Fe}/mL)^{-1}s^{-1}$<br>256.1 $mM_{Fe}^{-1}s^{-1}$
SAR_{Fe} (slope)	149 W/mg_{Fe}
SAR_{Fe}(@ 2.4 mg Fe/mL)	216 W/mg_{Fe}

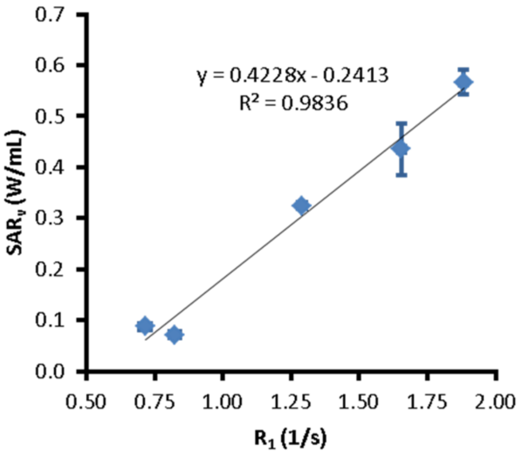

Figure S8. Relaxivity and SAR_{Fe} of sIONPs with a 16 nm shell are measured in VS55 and 1% agarose. The SAR_v is plotted as a function of R1, indicating a linear correlation between R1 and SAR_v. Calculations were determined with samples ranging from 0 to 4 mg Fe mL^{-1}.

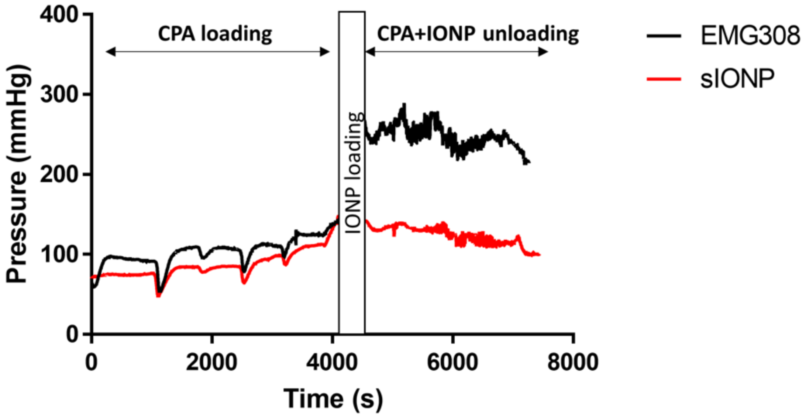

Figure S9. Kidney CPA loading and unloading prefusion pressure. The IONP loading pressure was not recorded. After loading EMG308, the pressure significantly increased due to the blockage of the vasculatures.

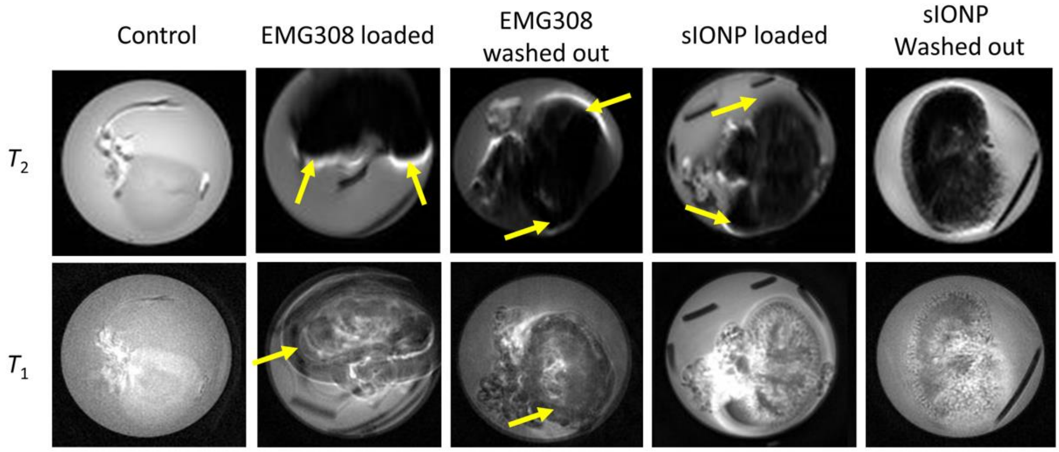

Figure S10. T_2- (spin echo, negative contrast) and T_1-weighted (MB-SWIFT, positive contrast) images of rat kidneys perfused with Euro Collins buffer (control), EMG308- and sIONP-loaded, and washed-out kidneys. Blurring, blooming, and pile-up artifacts (yellow arrow) indicate that the Fe concentration is higher than the imaging detection limit. All of the EMG308 images have distortion artifacts present. The T_2-weighted sIONP-loaded image has artifacts present. Due to the high Fe detection limit of SWIFT, the T_1 weighted sIONP-loaded image does not present artifacts. No artifacts were observed in the T_2 or T_1- weighted sIONP washed-out kidney images.
